# Chipper: discovering transcription-factor targets from chromatin immunoprecipitation microarrays using variance stabilization

**DOI:** 10.1186/gb-2005-6-11-r96

**Published:** 2005-11-01

**Authors:** Francis D Gibbons, Markus Proft, Kevin Struhl, Frederick P Roth

**Affiliations:** 1 Department of Biological Chemistry and Molecular Pharmacology, Harvard Medical School, Longwood Avenue, Boston, MA 02115, USA; 2 Instituto de Biología Molecular y Celular de Plantas (IBMCP), Universidad Politécnica de Valencia, Camino de Vera s/n, 46022 Valencia, Spain

## Abstract

Chromatin immunoprecipitation combined with microarray technology (Chip^2^) allows genome-wide determination of protein-DNA binding sites. The current standard method for analyzing Chip^2 ^data requires additional control experiments that are subject to systematic error. We developed methods to assess significance using variance stabilization, learning error-model parameters without external control experiments. The method was validated experimentally, shows greater sensitivity than the current standard method, and incorporates false-discovery rate analysis. The corresponding software ('Chipper') is freely available. The method described here should help reveal an organism's transcription-regulatory 'wiring diagram'.

## Abstract 2

A new method, implemented in software as 'Chipper', is described that allows genome-wide determination of protein-DNA binding sites from chromatin immunoprecipitation microarrays.

## Background

A major goal in understanding cellular behavior is to reveal the 'wiring' of transcriptional regulation, through which transcription factors (TFs) bind target-gene promoters to control gene expression. Promoter regions contain sequence elements - typically 5 to 12 nucleotides (nt) in length - at which TFs bind specifically. By enhancing/inhibiting transcription or recruiting complexes that remodel chromatin structure, TFs regulate expression of the genes whose promoters they bind. Chromatin immunoprecipitation (ChIP) is an experimental technique for identifying those regions of DNA bound by a particular protein, and is, therefore, a useful method for determining which genes have their promoters bound by a TF. In outline, the method consists of the following steps. The TF under study is crosslinked to DNA which is subsequently extracted and sheared into fragments approximately 400 nt long (1,000 nt resolution is usually sufficient to assign binding to the regulation of a specific gene, so it is rare to exceed this length [1]). The fragments are immunoprecipitated with an antibody specific to that TF (or to a peptide affinity tag fused to that TF), whereupon the crosslinks are reversed, the DNA precipitate amplified, and the intergenic regions (IGRs) containing the binding site(s) are determined by examining the relative abundance of each immunoprecipitated DNA fragment. The combination of ChIP with microarray technology is often called 'ChIP-chip' [1] and is referred to here as 'Chip^2^'. It has turned ChIP into a high-throughput technique for efficiently mapping gene regulatory networks [2-9].

Two-channel microarrays use hybridization to compare the abundance of specific nucleic acid sequences in one mixture to abundance of the same sequences in another control mixture. The choice of control mixture may greatly affect the outcome of the experiment. A typical choice is fragmented genomic DNA, which controls for the relative abundance and non-specific hybridization potential of genomic DNA fragments. Genomic DNA may be purified from 'whole-cell extract', which itself is sometimes used as a control. As some DNA fragments may be 'stickier' than others, a more stringent and laborious mock control (containing fragments recovered nonspecifically by immunoprecipitation (IP)) is sometimes performed, in which the TF does not have a fused affinity tag.

The change in abundance of a particular sequence between two mixtures is often measured in terms of 'fold-change' between the two channels (ratio) or, alternatively, the logarithm of fold-change (log-ratio). The IP channel serves as numerator, while the control is the denominator. The array surface between regions with spotted DNA is never completely 'dark', due to the combined effects of residual DNA fragments bound non-specifically to the array surface, and the experimentalist's control of the visual amplification ('gain') in the image analysis software. It is customary to subtract this 'background' from each spot because it reveals nothing about the protein-DNA binding. This subtraction raises the possibility, however, that the denominator could become negative or zero, in which case the log-ratio is not useful. Common strategies for handling zero or negative values are either to threshold or to discard data points altogether, neither of which is entirely satisfactory. A further, and perhaps more serious, problem is the practice of interpreting this fold-change as a measure of significance, when it provides no such statistical basis. Small random fluctuations in signals close to background, particularly in the denominator, are amplified, leading to spuriously high levels of 'fold-change' [10]. In other words, we should reduce our confidence in a twofold change between signals that are each near the background noise, compared to a twofold change between strong signals. Because we are generally more interested in whether a region is specifically bound at all than we are in the degree of its binding (occupancy), there is a need for an accurate measure of confidence in each measurement.

A statistical approach for analysis of mRNA abundance microarrays has been developed in which a 'single-array' error model accounts for variation in the background level for each microarray, while a 'gene-specific' error model describes variation of a single gene across replicate arrays. These two complementary models can be combined to estimate the error in each log-ratio measurement [10]. A variant of the single-array approach (in which there is gene-specific normalization) has been applied to transcription-factor binding site identification by means of Chip^2 ^in yeast [2]. Unfortunately, it requires one or more separate control experiments to determine error model parameters, in which identical nucleic acid mixtures are compared. This adds to the expense of the experiment; furthermore, error model parameters derived from a separate microarray are potential sources of systematic error, since quality can vary between microarrays.

## Results and discussion

Here we describe a new approach for assessing statistical significance of TF-binding from Chip^2 ^data. We illustrate our method using a Chip^2 ^analysis of Sko1 (also known as Acr1), a TF of the basic leucine zipper (bZIP) family (CREB sub-family) that regulates the expression of osmotic stress inducible genes [11-13]. We also use independent confirmation experiments of individual IGRs to validate our method.

### Combining replicates

We distinguish two kinds of repeated experiment. When the same IGR is spotted onto an array in more than one location, we term these measurements 'duplicates,' and we consider them as two spatially separated parts of the same 'spot'. Though other approaches have been described [14], for simplicity we average duplicate signals before analyzing them, giving us a single value that is less susceptible to physical blemishes on the slide. When the same IGRs are spotted onto two or more distinct microarrays, we term them 'replicates.' We consider each replicate as an independent measurement of the binding affinity or 'occupancy' of the IGRs.

### Variance stabilization

It is common to replicate genome-wide experiments several times, to improve confidence in the results, which may be degraded by array imperfections or by handling errors. Additional replicates can compensate for random error in individual measurements, and the typical number of replicates is likely to increase as the cost of microarrays falls [1]. Sometimes the most significantly enhanced IGRs are those with low signal-to-noise ratio, yet applying log-ratios to such signals has the potential to introduce many false positives because minor variations in a small denominator value can have a large effect on a ratio. A single-array error model can account for this variation in calculating significance for each IGR. The log-ratios themselves are difficult to interpret, however, because two IGRs with the same log-ratio may differ in significance, and a greater log-ratio does not indicate increased significance. An alternative approach, the method of variance stabilization, was described by two groups [15,16] and made available as part of the BioConductor project [17] in the package 'vsn' [15]. It uses a regression algorithm that is robust to outliers to scale and offset each channel independently, in such a way that the variance between channels is independent of signal strength. The transformation of the signal *y*_*i *_in the *i*th channel (*i *= 1 for IP, or *i *= 2 for control) can be expressed as:



where *α*_*i *_and *λ*_*i *_represent the background and noise in the *i*th channel, respectively. Because ln(*a*) - ln(*b*) = ln(*a*/*b*), the difference between the two transformed channels (Δ*h *≡ *h*_*i *_- *h*_2_) is then a generalized log-ratio that is asymptotically equivalent to the log-ratio of the original channels when both are high (*y*_*i *_>> *α*_*i*_), yet transforms smoothly to the difference between channels when both are low. This allows direct comparison between any two datapoints, even when they belong to opposite ends of the microarray's dynamic range. Two IGRs with the same *Δh *are equally significant, and greater *Δh *implies a more significantly bound IGR.

### Deriving error model parameters internally

Binding of protein to DNA is a dynamic, stochastic process in equilibrium. While every TF is likely to be bound to every IGR at least some fraction of the time, our goal here is to perform binary classification of the IGRs. We therefore consider IGRs to fall into two categories: those that are specifically bound by the TF and those that are not. We wish to compute a *p *value that expresses our degree of surprise at seeing a particular *Δh *score for a given IGR, under the null hypothesis that the IGR is not bound. The 'vsn' package can be used to variance-stabilize each array separately, or all of them simultaneously; we used the former method. Having computed the inter-channel variance-stabilized difference (*Δh*) for each spot, we may plot a histogram of all scores from a chip. We expect that most regions are not bound. Therefore, the distribution of *Δh *scores should be largely determined by random binding and measurement errors [18]. A smaller number of regions are bound, and those will tend to have positive scores, indicating higher occupancies in the IP channel than the whole-cell extract/mock control. Measurements in the negative portion of the *Δh *distribution should, therefore, be more completely dominated by unbound IGRs. By fitting a parametric curve to the region of the observed *Δh *distribution left of the mode, we obtain an estimate of the null distribution in the positive region of the *Δh *distribution. This is an essential feature of our method, because it allows us to estimate the distribution expected of unbound IGRs without performing an external control experiment in which an identical mixture is examined in both channels of a separate microarray. It is this null distribution that permits calculation of significance for each observed *Δh *value. The symmetric nature of the null distribution is an assumption of our model, and is based on our own experience and that of others [19].

**Figure 1:**
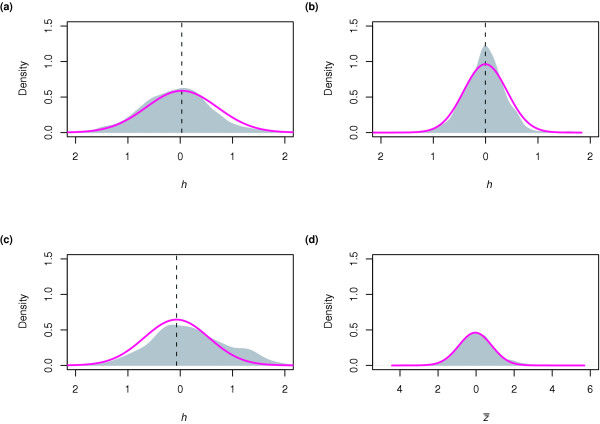
Three replicate two-channel Chip^2 ^experiments performed on Sko1 [22] were variance-stabilized. **(a-c) **Distributions of the *Δh *values obtained. Shaded gray areas indicate kernel-smoothed densities estimated from data. Magenta curves estimate the distribution of scores expected of unbound intergenic regions (IGRs) by fitting a normal distribution to the negative *Δh *side of the distribution. Sufficient statistics (mean, variance) of each fitted distribution are used to standardize the *Δh *distributions to a score *z*_*i *_for each replicate. **(d) **The distribution of the average score  over all three replicates. We computed a *p *value for each IGR under the null hypothesis that it is unbound, using the curve fitted to the negative portion of the empirical  distribution.

Specifically, a parametric distribution is fitted by minimizing the negative log-likelihood of the data to the left of the mode (found after smoothing the data using gaussian kernel-based density estimation) [20,21]. Three possible distributions were initially considered (normal, Cauchy, and Gumbel), but the normal distribution consistently obtains the best log-likelihood score. Goodness-of-fit for the fitted normal distributions was verified with a χ^2 ^test, and all passed with *p *< 10^-20^. The *Δh *scores from all replicates are standardized (centered to have zero mean and re-scaled to have unit variance) yielding a score *z*_*i *_= (*Δh*_*i *_- *μ*_*i*_)/*σ*_*i*_, where *μ*_*i *_and *σ*_*i *_represent the mean and standard deviation, respectively, of the *Δh *values obtained from replicate *i*. Figure 1a-c shows *Δh *distributions for three replicates [22]. We expect the distribution of *Δh *scores to be centered about zero; as shown by the vertical dotted lines in Figure 1, this is true to a very good approximation. Variance stabilization attempts to transform the data such that measurement error is uniform for each spot on a given array, and if replicate arrays were identical, one would expect to see the same variance in each array; large discrepancies between arrays might indicate problems with the quality of some of the arrays. Standardization is necessary to account for minor (on the order of 10%) differences in variance between arrays. Standardized scores are averaged to give an overall score (), the distribution of which is shown in Figure 1d. This distribution is again smoothed with a gaussian kernel, and fitted as described above. Finally, a *p *value for each IGR is computed on the  score, according to the null hypothesis that all IGRs are described by this fitted normal distribution, that is, they are not bound by the TF.

### Experimental verification of our dataset and evaluation of *p *value accuracy

**Figure 2:**
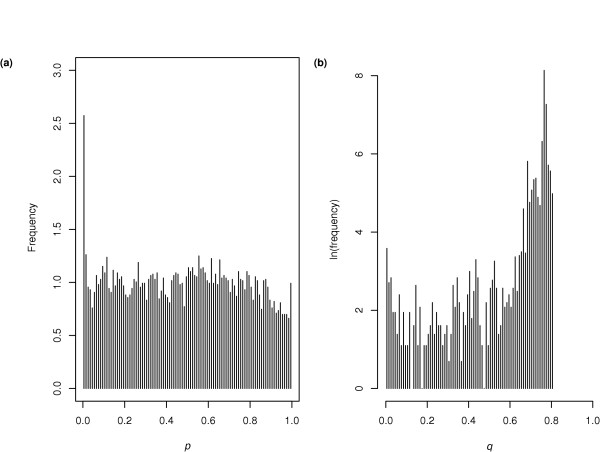
Observed distributions of *p *and *q *values. **(a) **The distribution of *p *values for the same data as in Figure 1. They are relatively uniformly distributed on the interval (0,1), except for a slight peak close to *p *= 0, indicating a small fraction of specifically bound intergenic regions (IGRs). **(b) **Corresponding *q *values, but with a log scale on the vertical axis. As one descends the ranked list of IGRs the *q *value rapidly approaches unity. That most IGRs have *q *close to 1 is expected given that the list of tested IGRs is long, and the number of true targets is generally small.

The distribution of computed *p *values is shown in Figure 2a. It clearly shows near-ideal behavior: uniform distribution across most of the interval (0,1) arising from the vast majority of unbound IGRs, and a peak close to *p = 0*, arising from bound IGRs. Figure 2b shows the distribution of *q *values. As expected, most IGRs have a high *q *value, consistent with the assumption that most are unbound. False discovery rates, as represented by *q *values [23], are particularly useful when the goal is discovery of TF-bound IGRs. For example, the *q *values for Sko1 (see Additional data file 1) indicate that scientists willing to accept a list of targets in which 33% are false positives should examine the top 224 entries using a more-accurate experimental method, while those only willing to tolerate a false-positive rate of 20% should restrict themselves to the top 91.

We independently validated 35 target genes spread widely across the top 350 in our list using targeted ChIP analysis. Considering only the 35 targets for which follow-up testing was performed, ranking of IGRs by the *p *values of Lee *et al*. [2] (see Additional data file 4) shows an ability similar to our method ('Chipper') at placing true positives above false positives. When considering all IGRs, however, there is little correlation between rank by our method and rank by the Lee *et al*. approach. In other words, top-ranking targets by one method are not top-ranking by the other. Thus, although our validation experiments are consistent with Chipper achieving the same sensitivity at a lower false-positive rate, it is also possible that the two methods are each adept at identifying different subsets of targets. The discrepancy may be due to some systematic error in determination of the parameters of the error model. As the error model parameters are not provided explicitly with their data, we could not investigate this possibility further. Inaccurate determination of error-model parameters can lead to unjustified confidence in differences based on noisy measurements. Therefore, in the task of ranking IGRs by the likelihood of being TF-bound, Chipper is on par and complementary to the Lee *et al*. approach and may outperform it. Furthermore, the Chipper algorithm uses an internally determined error model and thus is not subject to systematic errors that may arise via the separate control experiments required of the methods in Lee *et al*. [2]. Below we show that Chipper allows increased sensitivity at a given significance threshold.

Chip^2 ^experiments cannot distinguish the strand on which binding occurs, only the location at which it takes place. When binding is assigned to an IGR less than 2,000 nt in size, which happens to separate two genes on opposite strands, it is not possible to determine, on the basis of Chip^2 ^alone, which one is the target of a TF. For example, as illustrated in Table 1, *FAA1 *and *COT1 *are divergently transcribed genes separated by a 1,800 nt IGR. The IGR is split into *FAA1*-proximal and *COT1*-proximal IGR segments. The primers used for targeted ChIP (about 200 nt) are smaller than the sheared fragments used in the microarray experiments (500 nt), which gives them a greater spatial resolution. As the primers are designed for a specific promoter, and amplified by polymerase chain reaction, they are strand-specific. Only *FAA1 *is found to bind Sko1 in a targeted ChIP experiment, yet because both IGR segments overlap Sko1-bound fragments in the Chip^2 ^experiment, a spurious positive result is generated for *COT1*. We score correctly identified IGRs as true positives, even when only a single gene is verified in the targeted experiment. The Sko1 data, along with further study of Sko1 targets, are published elsewhere in the context of a focused study of Sko1 [22].

### False discovery rate analysis

**Figure 3:**
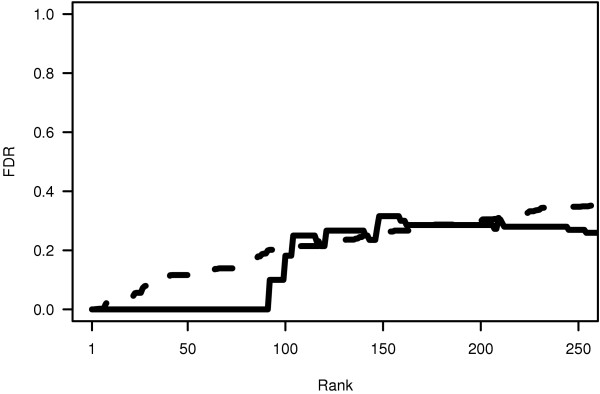
Agreement between predicted and empirical false-discovery rate for Sko1. The broken curve shows *q *values computed from the ranked list of *p *values, using QVALUE software [32]. The solid curve shows the false-discovery rate (FDR) computed using only targeted chromatin immunoprecipitation experiments (35 targets).

A common measure of significance used in hypothesis testing is the *p *value. In large-scale experiments like these, random chance can cause some IGRs to have *p *values that will be considered significant. Multiple hypothesis corrections (that is, corrections for the fact that a hypothesis is being tested multiple times, once for each IGR) are a popular approach in which the significance threshold is raised (or the *p *value lowered) as a function of the number of IGRs. Bonferroni-type [24] corrections are often conservative, in that many positives may be classified as non-significant ('false negatives'). This is borne out in our analysis of Sko1 Chip^2 ^data, in which, after multiple-hypothesis correction, only a small number of IGRs (<10) were significant, at an experimentwise *p *value = 0.05 or lower (equivalent to *p *= 1.06 × 10^-5 ^before multiple-hypothesis correction). However, the motivation of most Chip^2 ^users is not to cautiously establish a list of binding sites that are known with near-certainty. The attraction of Chip^2 ^is its high-throughput nature, which allows the experimentalist to rapidly generate a list of potential binding sites for subsequent study. A relatively recent alternative to the *p *value is the *q *value, which is a measure of false discovery rate (FDR) that has proven useful when the aim of an experiment is hypothesis generation rather than hypothesis testing [23,25,26]. Despite the fact that Chip^2 ^experiments are typically used for hypothesis generation, no previously reported analysis of Chip^2 ^experiments has employed an FDR approach. Figure 3 shows that the *q *values computed from our *p *values (broken line) agree quite well with our empirical FDR (solid line). As the first verified false positive ranks just above 100, our empirical FDR is zero to that point. Thereafter, it tracks the computed FDR quite closely until all true positives have been discovered.

### Validation with publicly available datasets

**Figure 4:**
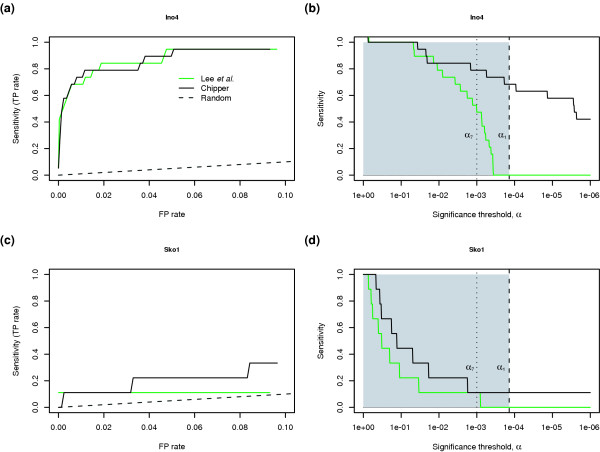
Performance of our algorithm on publicly available Chip^2 ^data [2] is evaluated using the Yeast Proteome Database collection of transcription factor targets [28,29] and compared with another popular means of computing *p *values [2]. **(a) **Receiver-operating characteristic curves for our method (black, 'Chipper') and that of Lee *et al*. [2] (green, 'Lee') using three replicate experiments for the transcription factor Ino4, made publicly available by Lee *et al. ***(b) **Sensitivity as a function of significance threshold. The broken line represents the performance of choosing potential targets at random. **(c,d) **Analogous curves for the transcription factor Sko1. FP, false positive; TP, true positive.

We obtained the raw data used by Lee *et al. *[2] and compared the *p *values produced by our algorithm with the published *p *values. The 7,200 IGRs were ranked using the appropriate score for each method, and the ranked lists were evaluated for the presence of targets annotated as bound by the TF of interest in the Yeast Proteome Database (YPD) [27,28]. Data for two TFs (Ino4 and Sko1) are shown in Figure 4, and analysis of another six TFs is shown in Additional data file 5. In Figure 4a we show the receiver-operating characteristic (ROC) curve for Ino4, which tracks the sensitivity of an algorithm (its ability to find true positives (TPs)) as a function of its tendency to turn up false positives (FPs). An optimal algorithm would rank all TPs at the top. Its ROC curve would begin at the lower left-hand corner (FP = 0, TP = 0), move vertically to the upper left-hand corner (FP = 0, TP = 1), and then across the top of the chart to the upper right-hand corner (FP = 1, TP = 1). As this is a hypothesis-generation technique, only those targets near the top of a ranked list are likely to be of interest; we therefore show only the region from FP = 0 to FP = 0.1. The ranking performance of each algorithm is good in this case, and there appears little to choose between methods: either one can achieve a sensitivity of almost 1.0 with a false-positive rate of about 0.05.

In practice, however, it is common to consider only those IGRs passing a standard threshold of significance (*p *< 10^-3 ^in Lee *et al*. [2] and Harbison *et al*. [8]). Therefore, we evaluated the same data, but rather than focusing on simple ranking ability, we examined the *p *value of each call (results for Ino4 shown in Figure 4b). We constructed the graph by choosing a significance threshold (*α*) and asking what fraction of the known true positives exceed the threshold (that is, have *p *values less than *α*). At *α* = 1, any algorithm will have perfect sensitivity because it calls all IGRs significant; this comes at the cost of specificity, as it is unable to distinguish between true and false positives. The *p *values reported by Lee *et al. *[2] are shown in green, those by our method are shown in black. The vertical dotted line indicates a threshold *α*_7 _= 10^-3 ^at which we would expect approximately 7 out of 7,200 intergenic regions to achieve significant scores purely by chance, even if none were bound by the TF. The vertical dashed line indicates the threshold *α*_1 _= 1.6 × 10^-4^, which we expect to be exceeded by chance for only one out of 7,200 IGRs. The unshaded area to the right of *α*_1 _indicates the region in which fewer than one IGR would be expected to exceed the threshold by chance. The higher an algorithm's sensitivity in this region (that is, the more true positives it puts here), the better. As we decrease the threshold, the sensitivity decreases slowly at first, for both methods. For the *p *values of Lee *et al. *[2], there is then a rapid reduction in sensitivity. At an *α* threshold such that only one false positive is expected, our method can recover more than half the known targets while Lee *et al*. [2] find none.

In Figure 4c, we show an ROC curve for the transcription factor Sko1, for which nine targets are annotated in the YPD. The error model of Lee *et al. *[2] ranks the targets slightly better than our method of average *z *scores. Yet, as shown in Figure 4d, for any given significance threshold, our algorithm returns more of those targets. Ino4 showed the most striking improvement in sensitivity (Figure 4b) for all TFs examined. However, for each of the eight TFs we examined (Figure 4 and Additional data file 5) our method called an equal or greater number of targets significant at the level of *α*_1 _than did the method of Lee *et al*. [2]. Thus, for all TFs examined, our method yields sensitivity either markedly better than or similar to that of the *de facto *standard method.

## Conclusions

We have developed a method for analyzing results from chromatin-immunoprecipitation/microarray (Chip^2^) experiments that computes *p *values without needing a separate control for developing a model of measurement error. The method proposed here successfully combines multiple replicates (separate arrays) and duplicates (same array) to produce a single overall *p *value for each IGR. By using variance stabilization rather than log ratios, we eliminate the need to threshold low-signal spots obtaining an alternative measure, *Δh*, which interpolates between a difference and a log-ratio and is monotonically related to significance. In addition, by averaging the resulting *z *score over replicates, an IGR that scores highly in a single replicate, but has no usable data in other replicates, may score well in the overall rankings. This is desirable in hypothesis generation: the algorithm should not be conservative, rather it should be sensitive and provide accurate *p *values by which the false positive rate can be judged. The *p *values produced by our algorithm behave as one would expect *p *values to: a broadly uniform distribution over the full range, but with enrichment near *p *= 0. Experimentalists can use the *q *values computed from these *p *values to generate a short list that is customized to their tolerance for false discoveries. We have evaluated our algorithm using the transcription factor Sko1 by performing targeted ChIP on 35 selected genes. Additionally, we have compared performance of our algorithm with that of a previous error model [2], using data from a public database of transcription-factor targets [28,29]. Generally, discrimination of true positives, as measured by ROC curves, is comparable for both methods. However, our method returns targets with more significant *p *values. We find that the observed false-discovery rate on these putative targets generally tracks that predicted by the *q *values, therefore validating the accuracy of the *p *values and *q *values produced by our method. To parameterize error models, the method presented here requires no external control microarray experiments (which may introduce systematic error), giving it a distinct advantage over others in current use. Software implementing the algorithm is available either in web-based form for online use, or for download by non-commercial users, from our website [30].

## Materials and methods

Chip^2 ^analysis on Sko1 was performed using three microarrays, each with duplicate spots. Genomic DNA was used as a negative control. We used targeted ChIP experiments on 35 putative targets of Sko1 to validate how well our algorithm finds TF binding sites. We selected targets distributed throughout the top-ranking 350 IGRs. Primers were specifically designed for each IGR, and each region was assayed three times both with and without the hemagglutinin (HA) epitope tag, and the results averaged. The *POL1 *open reading frame (ORF) and an ORF-free region were used as negative controls, since Sko1 is not expected to bind there. Each IGR was scored according to the ratio of its IP efficiency with the HA epitope tag compared to that of *POL1 *ORF (non-specific control). Based on prior experience, we chose a threshold of 2.0, above which we considered Sko1 to have bound to the IGR, and below which we considered it not to have bound. By this criterion, we found 21 bound IGRs, with the remaining 7 tested IGRs not bound. (The number of IGRs tested is less than the number of target genes because some IGRs are associated with more than one gene.) Of those scoring >2.0, we found that six (*ICY1*, *HOR7*, *YPR127W*, *DPM1*, *POS5*, and *RSN1*) also scored highly (above 2.0) without the tag, indicating that they bind non-specifically. In fact, only *POS5 *scored in the top 100 by our method. Further details on Chip^2 ^analysis of Sko1 and validation experiments are published elsewhere in the context of a focused study of Sko1 [22]. The complete dataset is available from the Gene Expression Omnibus (GEO) [31] under series accession number GSE3335.

## Additional data files

The following additional data are available with the online version of this paper. Additional data file 1 is a tab-delimited file containing the results of our analysis for all IGRs studied in our experiments. Additional data file 2 contains a detailed description of the comparison between the targets of Sko1 identified by Chipper when applied both to the data presented here and to other Chip^2 ^data [2], and previously published *p *values using a single-array error model [2]. Additional data files 3 and 4 are figures illustrating these comparisons. Additional data file 5 is a figure comparing the two methods as applied to results from six additional transcription factors. Additional data file 6 lists the IGRs identified as targets [29].

## Supplementary Material

Additional data File 1 A tab-delimited file containing the results of our analysis for all intergenic regions studied in our experiments. Click here for file

Additional data File 2 A detailed description of the comparison between the targets of Sko1 identified by Chipper when applied both to the data presented here and to other Chip^2 ^data [2], and previously published *p *values using a single-array error model [2]. Click here for file

Additional data File 3 A figure illustrating the comparisons made in Additional data file 2. Click here for file

Additional data File 4 A figure illustrating the comparisons made in Additional data file 2. Click here for file

Additional data File 5 A figure comparing the two methods described in Additional data file 2 as applied to results from six additional transcription factors. Click here for file

Additional data File 6 A list of the intergenic regions identified as targets [29]. Click here for file

## Figures and Tables

**Table 1:** Divergently transcribed genes, grouped in pairs of which at least one is a target of Sko1, according to a targeted ChIP assay

Gene	Promoter	Target?
*FAA1*	-827/-576	Yes
*COT1*	-1,743/-1,561	No
		
*PUT4*	-617/-372	Yes
*CIN1*	-1,007/ -	No
		
*RPI1*	-606/-451	Yes
*RHO3*	-1,611/-1,336	No
		
*SPO20*	-449/-211	Yes
*SOK2*	-1,896/-	No

Promoter distances are measured in nucleotides from the start codon of gene 1. Both genes of a pair are counted as positives in evaluating the algorithm described here, since distinguishing members of these pairs is beyond the resolution of Chip^2 ^experimental technology. ChIP, chromatin immunoprecipitation.
