# Supplementary material for: Chipper: discovering transcription-factor targets from chromatin immunoprecipitation microarrays using variance stabilization
Source: Genome Biol. 2005 Nov 1;6(11):R96. doi: 10.1186/gb-2005-6-11-r96 (PMC1297652; doi:10.1186/gb-2005-6-11-r96)
Supplement: Additional data File 2 — A detailed description of the comparison between the targets of Sko1 identified by Chipper when applied both to the data presented here and to other Chip2 data [2], and previously published p values using a single-array error model [2]. [file gb-2005-6-11-r96-S2.pdf]

Supplementary data for

Chipper: discovering transcription-factor targets from chromatin immunoprecipitation microarrays using variance stabilization, submitted by Francis D. Gibbons, Markus Proft, Kevin Struhl, and Frederick P. Roth.

In our main manuscript, we tested 35 putative targets, of which we verified 28 to be true targets of the transcription factor Sko1 ("true positives"), and verified the remaining 7 to be true non-targets ("false positives").

The single-array error model has been applied to $Chip^2$ studies as part of a large-scale effort to uncover transcription-factor targets. For each of 106 transcription factors, this study lists *p*-values for all 7200 intergenic regions (IGRs) tested. Overlap of the intergenic probe set used by Lee et al. with ours was incomplete, and only 22 of the 35 tested targets had corresponding probes in the Lee dataset. Our study had the dual goals of developing an internally controlled method for target discovery on the one hand, and discovering the true targets of Sko1 on the other, so we chose to test those putative targets that scored highly by our method. This represents an inherent selection bias in favor of our method, which we attempt to mitigate in the comparisons below, but which may nevertheless have affected comparisons based on these follow-up experiments.

Ranking the intergenic regions by increasing *p*-value, as supplied by Lee et al. [2], we find the verified targets are scattered widely over the top 5000 IGRs, as shown in Fig.S1. In an attempt to overcome the selection bias, we can ask how well each method performs the task of ranking the false-positives (verified non-targets) below the true-positives (verified targets). In this case, the discrepancy is much smaller, as shown in Fig.S2. For small false-positive rates (<0.1), our method appears to have somewhat higher sensitivity (i.e., at a given false-positive rate, it discovers a larger fraction of the known targets), though this effect is not large.

In addition to the lists of *p*-values, supplementary data from Ref. [2] also includes raw data for three $Chip^2$ replicates for Sko1. Running our algorithm on this dataset, we find its performance broadly comparable to its performance when applied to our own data, as shown in Fig. S2. Both datasets succeed in putting similar numbers of verified targets into the top 100, indicating that the principal difference between the two sets of results lies in the analysis method, not in the raw data.

Given our limited validation set, it is difficult to state conclusively that one method out-performs the other at ranking IGRs. Our method seems to be complementary, in that it identifies targets which escape detection in the single-array error model. Even in the event of comparable performance, the Chipper method requires no external control to determine error-model parameters. It therefore provides not only a reduction in the amount of work required to perform $Chip^2$ analysis, it also removes a potential source of systematic error.

Figure Legends:

Fig.S1. The fraction of true positives recovered is compared for two methods and two data sets: the single-array error model *p*-values of Lee et al. [2] (green), the Chipper method applied to the raw data supplied by Lee et al. (magenta), and the Chipper method applied to raw data generated by us [22] (cyan). Each dataset is scored relative only to the number of verified targets of Sko1 that were contained in the intergenic regions on the microarray.

Fig.S2. Relative ability of Chipper (green) and the single-array error model (magenta), at identifying targets of Sko1, both applied to the data of Lee et al [2]. Each algorithm is scored only on the targets for which a probe was included in the microarray experiment.

Fig.S3. ROC curves and sensitivity *vs.* significance threshold α for six TFs not shown in the main paper. Details as in Fig. 4.
