# Supplementary figures and images for: Chipper: discovering transcription-factor targets from chromatin immunoprecipitation microarrays using variance stabilization
Source: Genome Biol. 2005 Nov 1;6(11):R96. doi: 10.1186/gb-2005-6-11-r96 (PMC1297652; doi:10.1186/gb-2005-6-11-r96)

**Fig. 1 Gibbons, Proft, Struhl, and Roth**

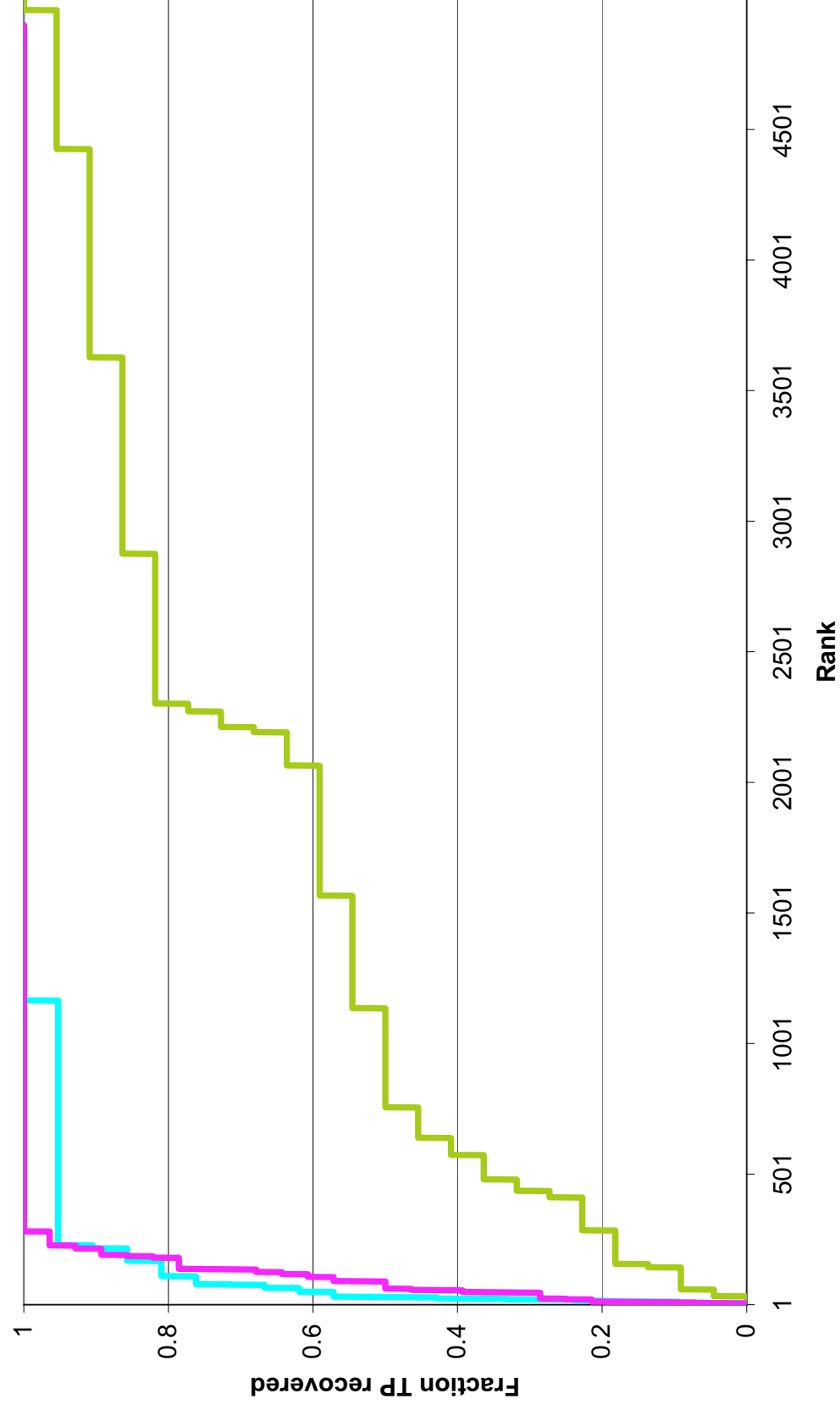

Supplement: Additional data File 3 — A figure illustrating the comparisons made in Additional data file 2. [file gb-2005-6-11-r96-S3.pdf]

**Fig. 2 Gibbons, Proft, Struhl, and Roth**

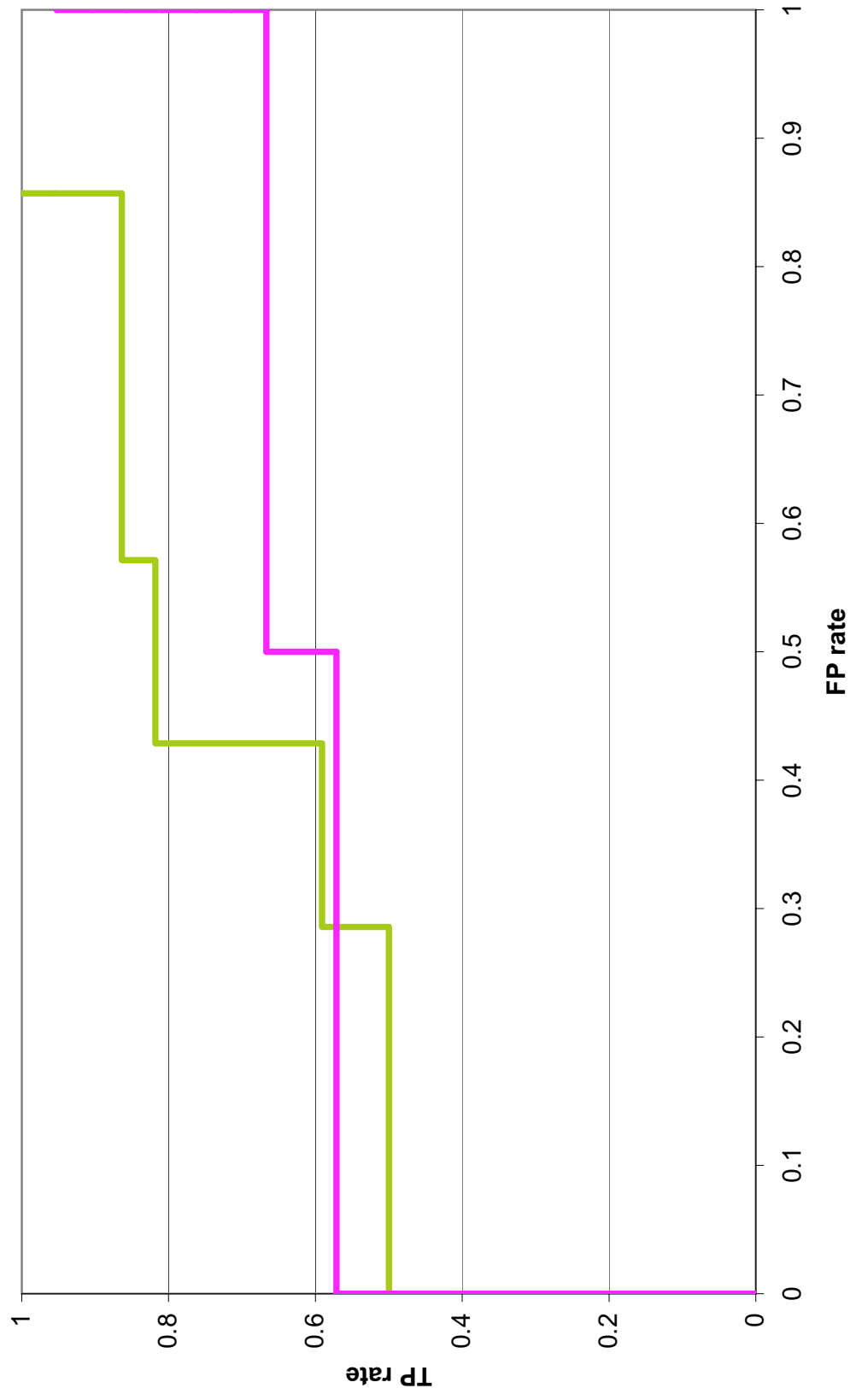

Supplement: Additional data File 4 — A figure illustrating the comparisons made in Additional data file 2. [file gb-2005-6-11-r96-S4.pdf]

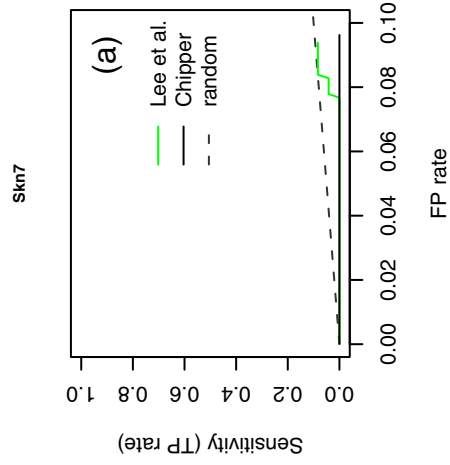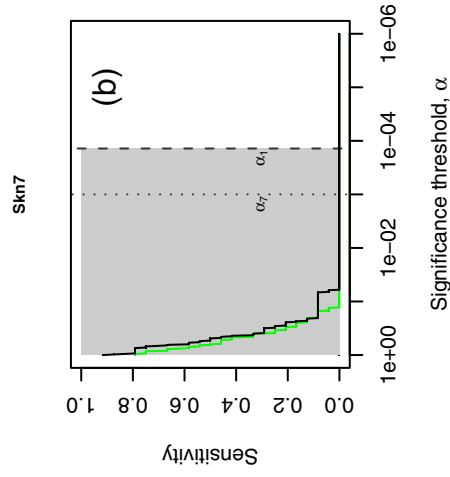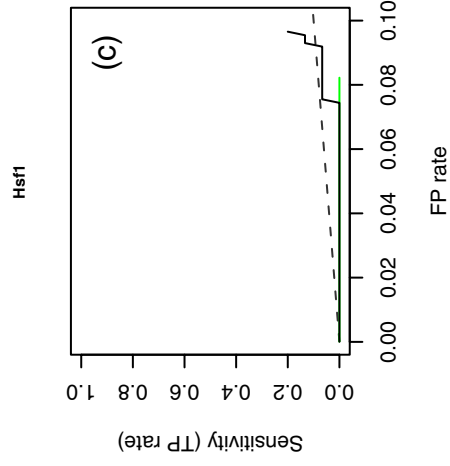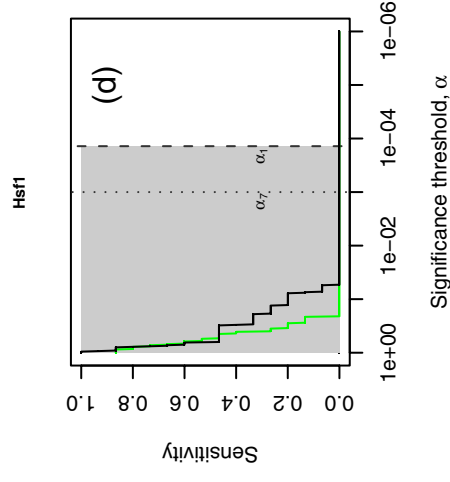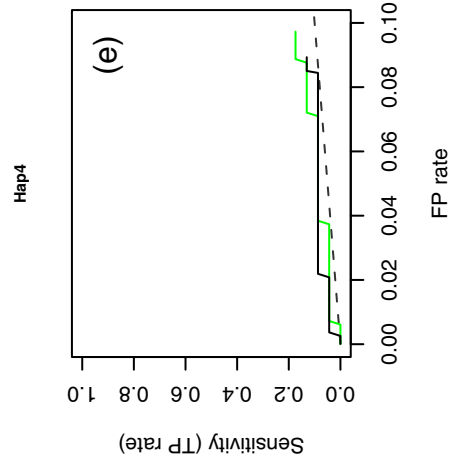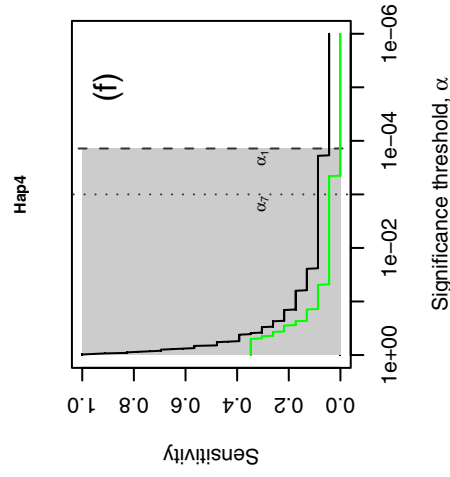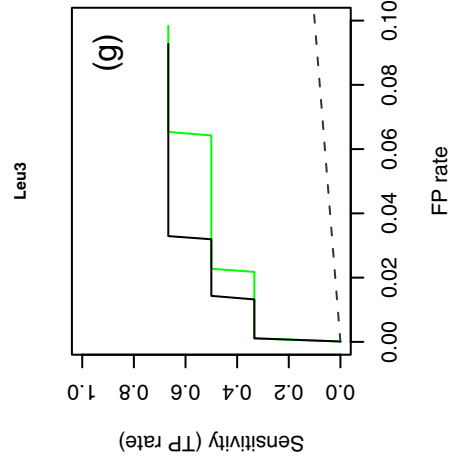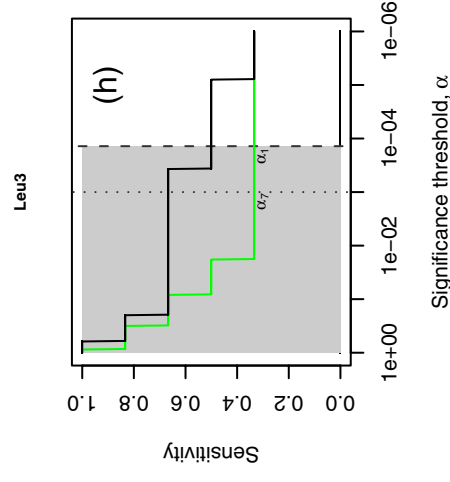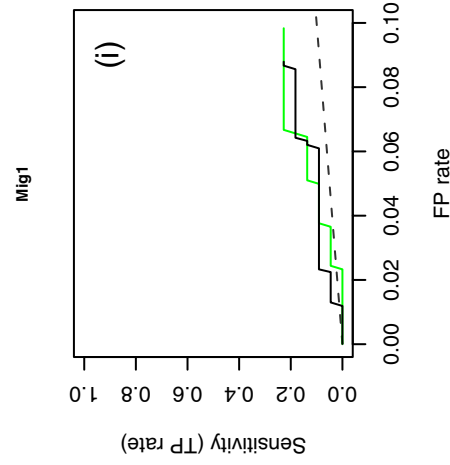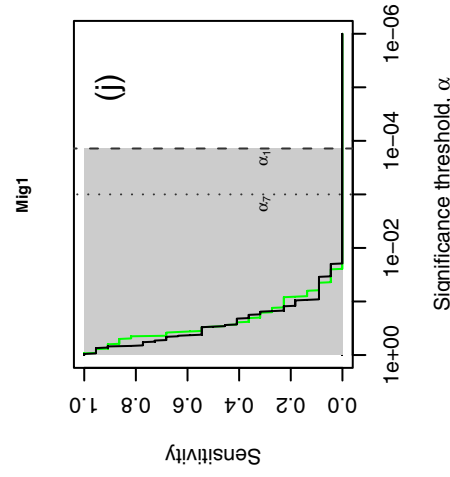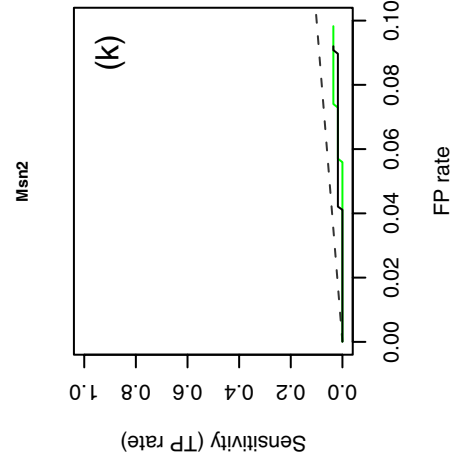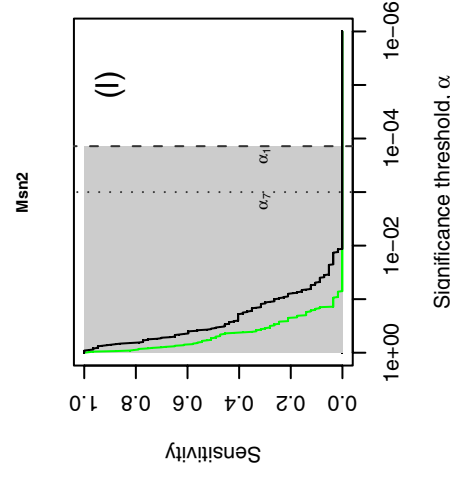

Significance threshold,  $\alpha$

FP rate

Significance threshold,  $\alpha$

Supplement: Additional data File 5 — A figure comparing the two methods described in Additional data file 2 as applied to results from six additional transcription factors. [file gb-2005-6-11-r96-S5.pdf]
